# Inverse Relationship between Serum VEGF Levels and Late In-Stent Restenosis of Drug-Eluting Stents

**DOI:** 10.1155/2017/8730271

**Published:** 2017-03-08

**Authors:** Jiasheng Yin, Li Shen, Meng Ji, Yizhe Wu, Sishi Cai, Jiahui Chen, Zhifeng Yao, Junbo Ge

**Affiliations:** ^1^Shanghai Institute of Cardiovascular Diseases, Zhongshan Hospital, Fudan University, Shanghai, China; ^2^Department of Cardiology, Zhongshan Hospital, Fudan University, Shanghai, China

## Abstract

Late in-stent restenosis (ISR) has raised concerns regarding the long-term efficacy of drug-eluting stents (DES). The role of vascular endothelial growth factor (VEGF) in the pathological process of ISR is controversial. This retrospective study aimed to investigate the relationship between serum VEGF levels and late ISR in patients with DES implantation. A total of 158 patients who underwent angiography follow-up beyond 1 year after intervention were included. The study population was classified into ISR and non-ISR groups. The ISR group was further divided according to follow-up duration and Mehran classification. VEGF levels were significantly lower in the ISR group than in the non-ISR group [96.34 (48.18, 174.14) versus 179.14 (93.59, 307.74) pg/mL, *p* < 0.0001]. Multivariate regression revealed that VEGF level, procedure age, and low-density lipoprotein cholesterol were independent risk factors for late ISR formation. Subgroup analysis demonstrated that VEGF levels were even lower in the very late (≥5 years) and diffuse ISR group (Mehran patterns II, III, and IV) than in the late ISR group (1–4 years) and the focal ISR group (Mehran pattern I), respectively. Furthermore, significant difference was found between diffuse and focal ISR groups. Serum VEGF levels were inversely associated with late ISR after DES implantation.

## 1. Introduction

Although the introduction of drug-eluting stents (DES) greatly reduced the incidence of in-stent restenosis (ISR), ISR remains a major complication after stent implantation. Recent data have reported that real-world patients with sirolimus-eluting stents have a 10.6% restenosis rate, while the rate of late ISR (defined as restenosis beyond one year) was much higher in patients with first-generation DES than in those with bare metal stents (BMS) [1]. Neoatherosclerosis was also more frequently observed after DES implantation than after BMS implantation, especially in patients with late restenosis or thrombosis [2]. These findings suggested that DES restenosis might have a different time course from that of BMS restenosis, which tends to occur within 1 year of implantation.

Given the significant implications of late restenosis in patients' prognostic outlook, it is of great clinical importance to identify which factors contribute to this process. A few studies conducted to date have identified that endothelial dysfunction and consequent neoatherosclerosis play a role in the development of late adverse events [3].

Vascular endothelial growth factor (VEGF) promotes endothelial cell function and stimulates endothelial cell migration and survival. Many animal studies have reported that VEGF accelerates endothelialization and inhibits neointima formation [4]. However, VEGF can also aggravate restenosis by influencing atherosclerotic plaque progression and inducing inflammation. Several studies have demonstrated that increasing levels of VEGF in the blood 24 hours [5] and 4 weeks [6] after percutaneous coronary intervention (PCI) were associated with restenosis. However, the angiographic follow-up duration of these studies was limited to 6–12 months. Habara et al. [7] demonstrated that the morphological characteristics of DES restenotic tissue among early (<1 year), late (1–3 years), and very late (≥3 years) phases of restenosis are different. Furthermore, in addition to the effect of increasing VEGF levels on restenosis after implantation, baseline VEGF levels were inversely associated with adverse cardiac events in one long-term follow-up study [8]. Thus, we speculated that the effect of VEGF on the formation of ISR after stent implantation differs over time. To the best of our knowledge, the relationship between circulating VEGF levels and late restenosis has not been previously investigated. Therefore, we evaluated serum VEGF levels in patients who underwent angiographic follow-up for more than 12 months after DES implantation, and we investigated the relationship between circulating VEGF levels and long-term ISR.

## 2. Methods

### 2.1. Study Population

We recruited 158 patients from a single center from December 2014 to June 2016. This retrospective study was approved by our hospital's Institutional Ethics Committee, and it was conducted in compliance with the Declaration of Helsinki. Informed consent was obtained from all participants.

Our population included patients with stable or unstable angina. Unstable angina was defined as chest discomfort suggestive of ischemia that was of new onset, that was increasing in severity, or that occurred at rest but without increased cardiac biomarkers [9]. All subjects underwent DES implantation more than 12 months before angiographic follow-up in our hospital. Patients were divided into two groups (ISR and non-ISR) per the results of their coronary angiography. Patients with a first ISR after drug-eluting stent implantation were categorized into the ISR group. ISR was defined as stenosis diameter ≥50% by visual estimation in the vessel segment within the stent or within 5 mm proximal or distal to the stent. In subgroup analysis, patients with ISR after 1 year were divided into two groups: late ISR (1–4 years: L-ISR) and very late ISR (≥5 years: VL-ISR); the median follow-up interval (5 years) was used to ensure that a comparable number of patients were allocated to each group. Follow-up intervals were calculated from the day of the index PCI procedure. Patients with ISR were further divided into three groups according to the Mehran morphological classification system for ISR [10]. The non-ISR group included patients without ISR or any other form of vessel revascularization. Clinical exclusion criteria included severe chronic heart failure (NYHA class III/IV), myocardial infarction including ST-segment elevation myocardial infarction (STEMI) and non-STEMI [11], severe concomitant valve disease, acute and chronic infections, autoimmune diseases, chronic renal insufficiency, malignancy, allergic diseases, use of anti-inflammatory or immunosuppressive drugs, and a recent (≤3 months) surgical procedure.

In this study, demographic and clinical data, including procedure age, gender, diabetes mellitus, hypertension, current smoking status, location of stent implantation, location of stent and stent restenosis, stent type, maximal stent diameter, stent length, angiographic follow-up duration, and percentage of left ventricular ejection fraction (LVEF%) on admission, were collected from in-hospital medical records and patient interviews.

### 2.2. Blood Samples

All patients provided a 5 mL venous blood sample prior to PCI. Blood samples were processed within one hour of collection. Peripheral blood was centrifuged at 2500 rpm for 10 minutes. Serum was collected and stored at −80°C for further experiments.

### 2.3. Laboratory Methods

Serum concentrations of VEGF were measured using a cytometric bead array (CBA) according to the manufacturer's protocol (BD Biosciences, San Jose, CA, USA) using a 50 *μ*L serum sample. Concentrations of serum cytokines were quantified using the CellQuest Pro and CBA software (Becton Dickinson, San Jose, CA, USA) and the Accuri C6 flow cytometer (BD Biosciences).

The serum high-sensitivity C-reactive protein (hs-CRP) level was detected by a modified laser nephelometric technique (Behring Diagnostics, GmbH, Marburg, Germany). Serum total cholesterol (TC), triglyceride (TG), high-density lipoprotein cholesterol (HDL-C), and low-density lipoprotein cholesterol (LDL-C) levels were determined by enzymatic methods using a Hitachi 7600 analyzer (Hitachi, Ltd., Tokyo, Japan).

### 2.4. Statistical Analysis

In patients with multiple ISR lesions, only the lesion with the highest rate of stenosis was included in the analysis. In the non-ISR group, lesions with the highest rate of stenosis during index PCI were included in the analysis. The data distribution was assessed according to the Kolmogorov-Smirnov test. Continuous variables were compared using unpaired Student's *t*-test or Mann–Whitney *U* test, as appropriate, and data were expressed as mean ± standard deviation or median and interquartile range, respectively. Categorical data were evaluated using the chi-square test. Multivariable logistic regression analysis model was used to study the risk factors associated with restenosis. The correlation between serum VEGF levels and clinical parameters was assessed using the Spearman rho test. All tests were two-sided, and a *p* value of 0.05 represented statistically significant differences. All analyses were performed using SPSS, version 21 (SPSS, Inc., Chicago, IL, USA).

## 3. Results

The subjects were divided into two groups as described in Section 2. Patients' baseline demographic and laboratory characteristics are shown in Table 1. Lesions and lesion stent characteristics are shown in Table 2. The median follow-up duration was 5 years. There were no significant differences between groups with respect to baseline characteristics including procedural age, gender, history of hypertension or diabetes mellitus, and current smoking status or laboratory values, including TC, TG, HDL, and LDL levels, but the hs-CRP level was higher in the ISR group compared to the non-ISR group (*p* = 0.05). There was no difference in the location of target vessel, number of stents implanted, and total stent length between two groups, but the mean maximal stent diameter was smaller in the ISR group. Focal ISR was found in 53.2% of patients, type II ISR in 15.2%, type III ISR in 16.5%, and type IV ISR in 15.2%.

**Figure 1:**
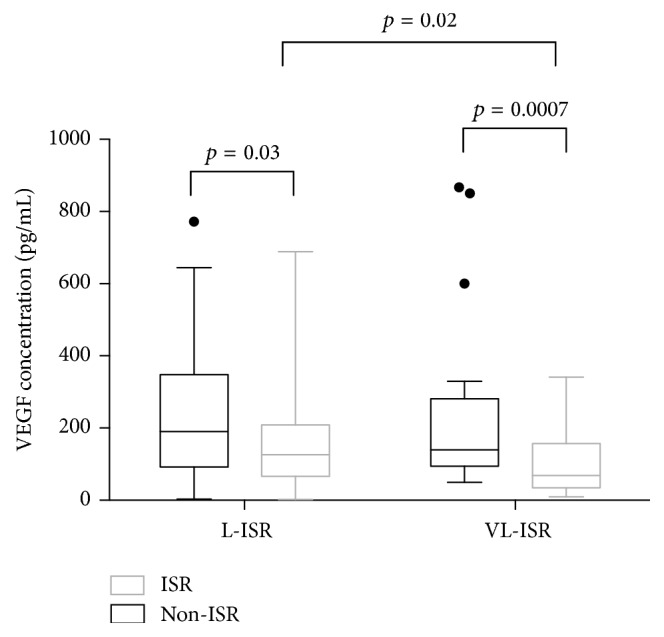
Comparison of serum VEGF levels between patients with ISR and without ISR in the late and very late phases. Data are presented as box plots with medians and interquartile ranges. *p* values were calculated using the Mann–Whitney *U* test. No significant difference was found between the L-non-ISR and VL-non-ISR groups. VEGF = vascular endothelial growth factor; ISR = in-stent restenosis; L-ISR = late in-stent restenosis; VL-ISR = very late in-stent restenosis.

**Figure 2:**
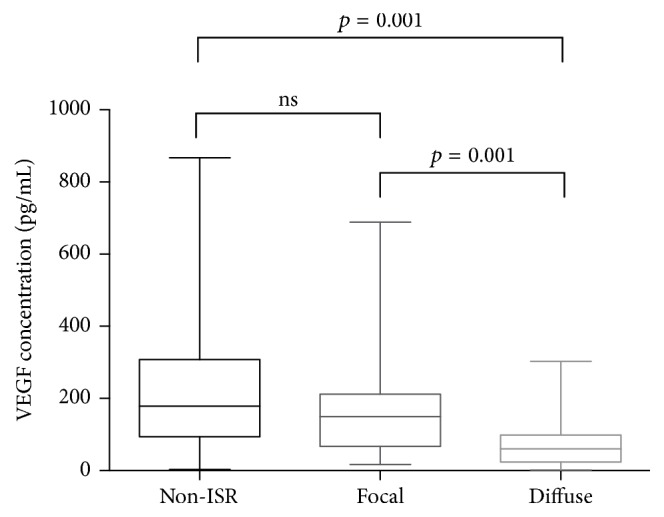
Comparison of serum VEGF levels among the non-ISR, focal, and diffuse ISR groups. Data are presented as box plots with medians and interquartile ranges. *p* values were calculated using the Kruskal-Wallis test and Mann–Whitney *U* test. VEGF = vascular endothelial growth factor; ISR = in-stent restenosis.

Serum VEGF levels were significantly lower in the ISR group than in the non-ISR group [96.34 (48.18, 174.14) versus 179.14 (93.59, 307.74) pg/mL; *p* < 0.0001] (Table 1). In the subgroup analysis, patients were divided into L-ISR, VL-ISR, L-non-ISR, and VL-non-ISR groups according to the follow-up intervals. We found that serum VEGF levels in both the L-ISR and the VL-ISR groups were significantly lower than those in the L-non-ISR and VL-non-ISR groups, respectively [L-ISR 125.99 (66.52, 181.68) versus L-non-ISR 190.48 (92.97, 345.5) pg/mL; *p* = 0.03 and VL-ISR 64.36 (35.71, 156.3) versus VL-non-ISR 139.95 (98.39, 271.4) pg/mL; *p* = 0.0007]. In addition, serum VEGF levels in the VL-ISR group were significantly lower than in the L-ISR group (*p* = 0.02) (Figure 1). In terms of angiographic patterns of restenosis, the level of serum VEGF in the diffuse ISR group was significantly lower than in the focal ISR and non-ISR group [60.49 (25.3, 106.95) versus 153.25 (70.82, 213.41) versus 179.14 (93.59, 307.74), respectively, *p* < 0.0001]; however, no significant difference was found between the focal ISR group and the non-ISR group (*p* = 0.06) (Figure 2).

To confirm the relationship among VEGF, conventional risk factors, and late ISR, several variables including procedure age, hypertension, diabetes mellitus, current smoking status, LVEF, serum creatinine, TC, TG, HDL-C, LDL-C, hs-CRP, maximal stent diameter, and total stent length were selected and analyzed using logistic regression analysis. We found that procedure age [OR = 1.055, 95% CI (1.012, 1.101), and *p* = 0.012], LDL-C [OR = 1.715, 95% CI (1.040, 2.827), and *p* = 0.034], and VEGF levels [OR = 0.914, 95% CI (0.914, 0.978), and *p* = 0.001] were independent predictors for the presence of ISR (Table 3). No significant correlations were found between VEGF levels and other indicators (Table 4).

## 4. Discussion

In the present study of patients who underwent angiographic follow-up for more than 1 year after DES implantation, we found that (1) serum VEGF levels were significantly lower in patients with a late ISR than in those without restenosis; (2) VEGF levels were significantly lower in very late ISR (≥5 years) and diffuse ISR groups than in the late ISR (1–4 years) and focal ISR groups, respectively; and (3) VEGF was an independent predictor of late ISR.

The contribution of VEGF to in-stent restenosis is controversial. Previous studies have demonstrated that increased levels of circulating VEGF at 24 hours and 4 weeks after PCI were closely related to ISR [5, 6]. The authors speculated that the increasing VEGF levels reflected a higher basal content of VEGF in the treated plaque and that VEGF levels within the arterial wall after vascular injury may promote inflammation [12] and angiogenesis [13], which accelerate neointima formation. However, other studies have demonstrated that endothelial injury after intervention was sufficient to stimulate neointima growth. VEGF promotes endothelialization and inhibits smooth muscle cell proliferation [14]. In addition, several animal studies have suggested that local VEGF gene transfer could improve endothelial healing and therefore reduce restenosis after stent implantation [4, 15]. The inconsistencies in the current literature highlight the importance of conducting further research on the role of VEGF and ISR.

In particular, previous studies were limited by the short duration of angiographic follow-up, which averaged 6 to 12 months in most studies. This follow-up period seemed insufficient in consideration of mounting evidence indicating that late in-stent restenosis beyond 1 year appeared to be more common in the first-generation DES era compared to that of the BMS era [1, 16, 17]. The exact mechanism contributing to this difference has not yet been elucidated, but many studies have suggested that late ISR in DES and BMS differs with respect to pathogenesis and histopathologic features [18]. Furthermore, the incidence of neoatherosclerosis, which was another important indicator of late ISR, was much greater in first-generation DES compared to that in BMS [19] and also occurs much earlier [2]. Late adverse events have also been attributed to poor reendothelialization in addition to inflammatory and hypersensitivity reactions [4]. In our study, we focused exclusively on ISR beyond 1 year and found lower serum VEGF levels in the late ISR group compared to that in the non-ISR groups. Furthermore, there was a significant difference between the VL-ISR and L-ISR groups. We speculated that lower VEGF levels might reflect endothelial dysfunction in late ISR patients, which is consistent with previous studies that have demonstrated an association between VEGF and endothelial healing and function [20, 21]. Ramos et al. [8] reported that low VEGF concentration was associated with an increased risk of hospitalization and combined adverse cardiac events in a 5-year follow-up study. Nakata et al. [22] demonstrated that the VEGF level in the anterior interventricular vein was significantly lower than that in the aortic root and was associated with impaired coronary endothelial function in patients implanted with a first-generation DES. The incidence of delayed endothelial healing and further neoatherosclerosis, which causes late adverse events, was higher in patients with endothelial dysfunction; this might explain the negative correlation between VEGF concentrations and the onset of late ISR. The contrary results on the relationship between VEGF levels and ISR also suggested that VEGF plays multiple roles in the formation of restenosis.

Interestingly, we observed that VEGF was associated not only with the development of ISR, but also with the pattern of ISR, as we demonstrated that VEGF levels in patients with diffuse ISR were significantly lower than that in those with focal ISR. This finding is significant because several previous studies have demonstrated that the prognosis of focal and diffuse ISR differs [23, 24]. These studies, in combination with our own, suggest that a different underlying mechanism contributes to the development of focal and diffuse ISR. Further studies are needed to investigate the exact mechanism of the formation of different ISR patterns, and the role that VEGF plays in it.

This study has several limitations. First, this was a retrospective study and therefore we were unable to establish a causal relationship between low VEGF levels and late ISR development. Serial measurements may provide a clearer understanding of this relationship. Second, the study population was relatively small and recruited from a single center, thus limiting the statistical power of the study. Finally, we used angiography to investigate patients with restenosis, instead of more advanced technologies such as intravascular ultrasound or optical coherence tomography that could have provided us with more detailed information.

## 5. Conclusions

Serum VEGF levels were significantly lower in patients with ISR beyond 1 year than in non-ISR patients. VEGF levels in the VL-ISR group (≥5 years) were lower than those in the L-ISR group (1–4 years) and non-ISR group. Finally, VEGF levels were significantly lower in patients with diffuse ISR than in those with focal ISR, while no significant differences were found between the focal ISR and non-ISR groups. Procedure age, LDL-C, and VEGF levels were independent risk factors for ISR.

## Figures and Tables

**Table 1:** Baseline and laboratory characteristics of the study population.

Characteristic	ISR (*n* = 79)	Non-ISR (*n* = 79)	*p* value
Procedure age, years	64.57 ± 9.26	61.62 ± 10.18	0.06
Male gender, *n* (%)	66 (83.5)	69 (87.3)	0.23
Hypertension, *n* (%)	52 (62.5)	53 (67.1)	0.86
Diabetes mellitus, *n* (%)	32 (40.5)	26 (32.9)	0.32
Current smoker, *n* (%)	19 (24.1)	18 (22.8)	0.85
LVEF (%)	61.74 ± 7.31	63.21 ± 5.87	0.17
Creatinine (*μ*mol/L)	79.36 ± 24.18	80.77 ± 25.27	0.72
TC (mmol/L)	3.30 (3.01, 4.07)	3.37 (2.73, 4.10)	0.43
TG (mmol/L)	1.22 (0.96, 1.74)	1.37 (0.91, 2.03)	0.37
LDL-C (mmol/L)	1.86 ± 0.86	1.62 ± 0.77	0.07
HDL-C (mmol/L)	1.00 ± 0.33	1.01 ± 0.38	0.93
hs-CRP (mg/L)	1.3 (0.3, 4.0)	0.5 (0.17, 1.65)	0.05
VEGF (pg/mL)	96.34 (48.18, 174.14)	179.14 (93.59, 307.74)	<0.01

Values are presented as number (%), mean value ± standard deviation, or median (interquartile range). LVEF = left ventricular ejection fraction; hs-CRP = high-sensitivity C-reactive protein; VEGF = vascular endothelial growth factor; TC = total cholesterol; TG = total triglyceride; LDL-C = low-density lipoprotein cholesterol; HDL-C = high-density lipoprotein cholesterol.

**Table 2:** Stent and ISR vessel characteristics of the study population.

	ISR (*n* = 79)	Non-ISR (*n* = 79)	*p* value
Target vessel			
LM	3 (3.0)	2 (2.5)	0.59
LAD	42 (53.2)	40 (50.6)	
LCX	12 (15.2)	10 (12.7)	
RCA	22 (27.8)	27 (34.2)	
Number of stents implanted (*n*)	1.49 ± 0.57	1.58 ± 0.65	0.47
Total stent length (mm)	41.36 ± 18.4	42.46 ± 21.04	0.96
Maximal stent diameter per lesion (mm)	2.97 ± 0.36	3.14 ± 0.39	0.02
ISR Mehran classification, *n* (%)			
Type I	42 (53.2)	N/A	N/A
Type II	12 (15.2)	N/A	N/A
Type III	13 (16.5)	N/A	N/A
Type IV	12 (15.2)	N/A	N/A

Values are presented as number (%) or mean value ± standard deviation. LM = left main artery; LAD = left anterior descending artery; LCX = left circumflex artery; RCA = right coronary artery; ISR = in-stent restenosis; N/A = not applicable.

**Table 3:** Multivariate binomial regression analysis to study risk factors related to in-stent restenosis.

Variables	OR	95% CI	*p* value
Procedure age, year	1.055	1.012–1.101	0.012
VEGF (pg/mL)	0.945	0.914–0.978	0.001
LDL-C (mmol/L)	1.715	1.040–2.827	0.034

OR = odds ratio; CI = confidence interval; VEGF = vascular endothelial growth factor; LDL-C = low-density lipoprotein cholesterol.

**Table 4:** Correlation analysis between VEGF and baseline characteristics.

	*r*	*p* value
Procedure age (years)	0.08	0.33
Gender (%)	−0.08	0.35
Current smoker (%)	−0.07	0.41
Hypertension (%)	−0.06	0.49
Diabetes mellitus (%)	−0.10	0.21
LVEF (%)	0.08	0.34
Serum creatinine (*μ*mol/L)	−0.02	0.79
TC (mmol/L)	0.10	0.24
TG (mmol/L)	0.08	0.35
HDL-C (mmol/L)	0.04	0.66
LDL-C (mmol/L)	0.08	0.35
hs-CRP (mg/L)	0.03	0.70
Number of stents per lesion (*n*)	0.14	0.07
Total stent length (mm)	0.07	0.38
Maximal stent diameter (mm)	−0.01	0.88

LVEF = left ventricular ejection fraction; hs-CRP = high-sensitivity C-reactive protein; VEGF = vascular endothelial growth factor; TC = total cholesterol; TG = total triglyceride; LDL-C = low-density lipoprotein cholesterol; HDL-C = high-density lipoprotein cholesterol.
